# Oral and upper gastrointestinal Crohn’s disease: new perspectives on pathogenesis and clinical management

**DOI:** 10.3389/fcell.2026.1907519

**Published:** 2026-08-21

**Authors:** Xianhong Shi, Beibei Wang, Xiaohui Zhao, Heng Li, Tianqi Zhou, Bin Li, Lulu Tian, Kai Yin

**Affiliations:** 1 Department of General Surgery, Affiliated Hospital of Jiangsu University, Jiangsu University, Zhenjiang, Jiangsu Province, China; 2 Department of Pathology, Affiliated Hospital of Jiangsu University, Jiangsu University, Zhenjiang, Jiangsu Province, China; 3 Department of General Surgery, Affiliated Hospital of Jiangsu University, Institute of Digestive Diseases, Jiangsu University, Zhenjiang, Jiangsu Province, China

**Keywords:** biomarkers, crohn’s disease, diagnosis, oral involvement, treatment, underlying mechanism, upper gastrointestinal

## Abstract

Crohn’s disease is a chronic inflammatory bowel disease that is closely associated with genetic factors, immune dysregulation and microbial imbalance. Lesions involving the oral cavity and upper gastrointestinal tract constitute a common yet clinically overlooked manifestation of this disease. This article reviews the cellular and molecular mechanisms underlying disease onset and progression, and evaluates the diagnostic performance of various noninvasive and serum biomarkers. It also summarizes multimodal diagnostic approaches and corresponding therapeutic strategies based on current clinical practice. Currently, relevant clinical trials and standardized evaluation criteria remain lacking, largely because of the limited understanding of disease pathogenesis. Therefore, high-quality basic and translational research will be essential to deepen the understanding of this disease and facilitate the development of precision diagnostic and therapeutic strategies.

## 1 Introduction

Crohn’s disease (CD) is a complex, lifelong inflammatory bowel disorder characterized by discontinuous, transmural inflammation that can affect the entire alimentary canal. Although the ileocolonic region is the most commonly affected site, CD can also involve the upper gastrointestinal (UGI) tract, a manifestation that profoundly influences disease behavior, therapeutic strategies, and patient prognosis (Greuter et al., 2018).

Since Gottlieb’s initial report in 1937, upper gastrointestinal Crohn’s disease (UGI-CD), once considered rare, has become increasingly recognized, although its reported prevalence of 0.3%–5% in adults likely underestimates its true frequency. This underestimation stems from several factors, including subtle or overlapping symptoms with more common conditions like gastroesophageal reflux disease, the historical lack of routine endoscopic evaluation of the UGI tract in adults with CD, and the patchy or submucosal nature of the inflammation, that may evade detection by superficial biopsies (Moon et al., 2020; Laube et al., 2018). In accordance with the Vienna classification, UGI involvement (L4) is defined as involvement of a segment proximal to the distal ileum. The subsequent Montreal Classification designates UGI involvement (L4) as a disease modifier that can coexist with any of the primary disease locations, including terminal ileal (L1), colonic (L2), and ileocolonic (L3) involvement (Satsangi et al., 2006).

Oral, esophageal, gastric, or duodenal involvement has been reported in up to 5% of patients with symptomatic CD. However, the epidemiological and clinical characteristics of UGI-CD differ substantially in pediatric populations, in which the prevalence is notably higher. This discrepancy is largely attributable to standardized pediatric diagnostic protocols that mandate initial UGI endoscopy with biopsies, suggesting that many adult cases may remain undetected until complications develop (Yuan et al., 2024; Pimentel et al., 2019). Greuter et al. reported UGI involvement in 6.5% of patients at diagnosis, with an additional 13.1% developing it during follow-up, indicating that its frequency increases over time. The same study identified male sex and age of ≤16 years as predictors of this involvement pattern (Greuter et al., 2018). The Third European Evidence-Based Consensus on the Diagnosis and Treatment of CD recommends further evaluation to determine the site and extent of CD, irrespective of colonoscopy findings (Gomollón et al., 2017).

Although UGI-CD has historically been described according to anatomical location, accumulating evidence supports the existence of shared pathogenic mechanisms across the oral cavity, esophagus, and gastroduodenal tract. These mechanisms include: (1) genetic susceptibility, with NOD2/CARD15 gene polymorphisms associated with UGI-CD lesions (Ingle et al., 2015; Zbar et al., 2012); (2) aberrant Th1-predominant adaptive immune responses associated with Anti-Saccharomyces cerevisiae antibody (ASCA) positivity in patients with CD (Ingle et al., 2015; Zbar et al., 2012; Ribaldone et al., 2020); (3) site-specific microbial dysbiosis that perpetuates mucosal inflammation (Elzayat et al., 2023; Uchida et al., 2024; Targan et al., 2005). Although these mechanisms are shared, their differential expression across anatomical segments of the digestive tract results in distinct clinical and pathophysiological features, which are discussed in the following sections.

Building on the shared pathogenic mechanisms discussed above, this review aims to examine the clinical presentations across different UGI segments, critically evaluate current and emerging diagnostic modalities, and synthesize the available evidence on medical, endoscopic, and surgical therapies. By integrating mechanistic insights with clinical translation, this review seeks to bridge existing knowledge gaps and provide a more comprehensive perspective on the diagnosis and treatment of UGI-CD.

## 2 Oral Crohn’s disease

Although CD primarily affects the intestines, it can also manifest with extraintestinal manifestations. Oral cavity involvement is termed Oral Crohn’s Disease (OCD) (Rowland et al., 2010). In 1969, Dyer and colleagues were among the first to identify and describe oral lesions in two patients with CD (Dyer et al., 1969). In the same year, Dudeney reported another patient with CD who presented with oral symptoms (Dudeney and Todd, 1970). Subsequently, numerous cases of oral involvement in CD have been reported.

Despite detailed descriptions and classification systems, reported prevalence rates vary widely, ranging from as low as 5% to more than 50% across studies, with most publications reporting rates between 20% and 50%. This variation is likely influenced by demographic factors, including patient age, sex, and ethnicity, as well as by whether the diagnostic assessment included evaluation by an oral health specialist (Rowland et al., 2010; Lankarani et al., 2013). Oral lesions are notably more common in pediatric populations than in adults, with one study reporting a prevalence of up to 48%, predominantly among males (Pittock et al., 2001). Furthermore, OCD typically presents at a younger age than CD overall and is more prevalent among patients with proximal gastrointestinal or perianal disease (Lankarani et al., 2013; Pittock et al., 2001).

### 2.1 Manifestation

The lips are the most common site of oral lesions in CD, where the lower lip is often more affected than the upper lip, followed by the buccal mucosa and gingiva (Katsanos et al., 2015). The manifestations of OCD include both specific and nonspecific lesions, and these oral findings may precede, coincide with, or follow the onset of intestinal disease. Specific lesions include a pebbly appearance of the oral mucosa, indurated tag-like lesions, deep linear ulcers (usually located in the buccal vestibule with hyperplastic folds), mucogingivitis, lip swelling with vertical fissures, and a midline fissure (Table 1). Nonspecific lesions may result from chronic inflammation, malnutrition, and medication-related adverse effects and include aphthous stomatitis, periodontal disease, angular cheilitis, pyostomatitis, glossitis, and lichen planus (Muhvić-Urek et al., 2016; Živić et al., 2023).

**TABLE 1:** OCD specific lesions.

Specific oral lesions	Vulnerable areas	Paraphrase
Cobblestone appearance	Posterior buccal mucosa	Hyperplastic swelling in buccal fissures leads to nodular mucosal bulging (Laube et al., 2018; Ribaldone et al., 2020)
Indurated tag-like lesions	Buccal mucosa, vestibule, or retromolar areas	Noncaseating granulomas occur in 75% of biopsies (Lankarani et al., 2013; Agi et al., 2022)
Deep linear ulcers	Buccal sulci	Usually accompanied by proliferative folds (Laube et al., 2018; Muhvić-Urek et al., 2016)
Mucogingivitis	The entire gingiva	Swelling, hyperplasia, and granularity ± ulceration (Ribaldone et al., 2020; Lankarani et al., 2013)
Swelling of the lips with vertical fissures	-	Persistent diffuse swelling of the lips/buccal mucosa (soft or firm), with lip fissures often colonized by microorganisms (Lankarani et al., 2013; Muhvić-Urek et al., 2016)
Median lip fissure (Lankarani et al., 2013)	-	-

This table shows the specific manifestations, common locations, and descriptions of oral lesions.

Nonspecific lesions are more common than specific lesions, making the differential diagnosis particularly challenging. Aphthous stomatitis is characterized by shallow, circular ulcers with a central fibrinous exudate and erythematous margins. The clinical features of angular cheilitis include erythema, with or without painful fissures, and oral ulceration. These clinical manifestations may result from anemia or a fungal or bacterial infections. Pyostomatitis is more frequent in ulcerative colitis (Trost and McDonnell, 2005).

Periodontal disease is a chronic inflammatory condition affecting the supporting tissues of the teeth and is initiated by bacterial biofilms that adhere to the tooth surface. In its early stages, periodontal disease manifests as gingivitis. Without timely intervention, inflammation may progress to periodontitis, resulting in damage to the periodontal ligament and alveolar bone. Research findings reported by Miloš Živić indicate that 47.4% of patients with CD in the study had comorbid periodontal disease, demonstrating a correlation between CD activity and deterioration of the periodontal supporting tissues. Patients with active inflammatory bowel disease (IBD) exhibited greater periodontal disease severity and more advanced periodontal disease stages than those in remission. Nevertheless, given the limited sample size, insufficient demographic representation, and restricted scope of the measured variables, additional well-designed prospective controlled studies are warranted (Živić et al., 2023).

Orofacial granulomatosis (OFG) is difficult to distinguish from OCD on both clinical and pathological aspects, and the precise association between OFG and gastrointestinal CD remains unclear. OFG has been reported as an early manifestation of CD. A substantial proportion of patients with OFG subsequently develop symptomatic gastrointestinal CD. Therefore, patients with OFG may subsequently develop CD (Zbar et al., 2012; Rowland et al., 2010). In 1985, Gibson et al. used the term OFG to define a group of OCD with no evidence of CD (Gibson et al., 2000).

Granulomatous cheilitis is the most common manifestation of OFG. The lips are the most commonly affected site, and the condition may occur alone or in combination with other manifestations. Lip swelling commonly results in painful vertical fissures (Lankarani et al., 2013). OFG is characterized by three types of ulcerations: deep oral ulcers with hyperplastic peripheral mucosa, painful ulcers, and small abscesses, typically located along the gingival margin or soft palate (Zbar et al., 2012). In the largest study of OFG, the mean age at onset was 20 years, with no sex predilection. The pathogenesis is not fully understood but is believed to involve allergic, infectious, and genetic factors (Lankarani et al., 2013).

A rare form of OFG in adults is Melkersson-Rosenthal syndrome, which is characterized by the classic triad of oro facial swelling, intermittent facial paralysis and lingual fissuring (Lankarani et al., 2013). In addition, systematic observational studies in pediatric patients with OFG suggest that eliminating certain predisposing factors (such as cassia bark aldehyde, benzoate additive, carnosine, monosodium glutamate, cocoa and sunset yellow), may effectively improve oral lesions (Endo and Rees, 2007; White et al., 2006).

### 2.2 Diagnosis

Because oral lesions may occur before the onset of IBD, IBD should be considered in patients presenting with oral symptoms (Harikisha et al., 2012). Therefore, collaboration between oral medicine specialists and gastroenterologists is essential for the early diagnosis of CD.

Blood tests include “traditional” non specific inflammatory parameters (erythrocyte sedimentation Rate, C-reactive protein). Among anti-neutrophil cytoplasmic antibodies (ANCA) and ASCA are the most commonly used to diagnose and classify the prognosis of CD patients (Hedin et al., 2019). The indirect immunofluorescence assay for ANCA was used to detect two basic ANCA patterns: cytoplasmic anti-neutrophil cytoplasmic antibodies (C-ANCA) and perinuclear anti-neutrophil cytoplasmic antibodies (P-ANCA). Atypical P-ANCA, which is most frequently detected in patients with IBD, is present in 40%–80% of patients with UC and less frequently in CD (5%–25%) (Papp et al., 2007). ASCA specifically related to CD, sensitivity of 40%–60% and specificity of 80%–90%, However, it is crucial to critically appraise the distinct diagnostic value of these systemic markers in the context of isolated oral lesions, as opposed to their established role in evaluating patients with suspected intestinal CD. In patients with established intestinal CD, ASCA positivity may support the diagnosis and correlate with a more aggressive phenotype. Conversely, for a patient presenting with oral lesions suspicious for OCD but without any gastrointestinal symptoms, the clinical utility of ASCA is limited (Ribaldone et al., 2020; Sandborn et al., 2001). Some studies have identified and preliminarily evaluated three novel antiglycan antibodies as serum biomarkers: anti-laminaribioside carbohydrate antibodies (ALCA), anti-chitobioside carbohydrate antibodies (ACCA), and anti-mannobioside carbohydrate antibodies (AMCA). Several investigators evaluated the diagnostic accuracy of these tests and reported that gASCA and AMCA antibody titers demonstrated the highest sensitivity in patients with CD (Table 2) (Simondi et al., 2008; Malickova et al., 2010), but their role in isolated OCD has not been established.

**TABLE 2:** Overview of diagnostic biomarkers.

Biomarker categories	Biomarker	Specimen type	Sensitivity	Significance
Serological marker	ASCA	Serum	40%–60%	It is the most widely used serological marker associated with CD. Positivity may be related to a Th1-type immune response (Ribaldone et al., 2020; Sandborn et al., 2001).
	ANCA	Serum	UC: 40%–80%	It is mainly used for the differential diagnosis of CD and UC. The positivity rate is low in CD (Papp et al., 2007).
			CD: 5%–25% (p-ANCA)	
	ALCA	Serum	25%	As a novel biomarker, it may enhance the diagnostic sensitivity for CD, especially in ASCA-negative patients (Malickova et al., 2010).
	ACCA	Serum	27%	
	AMCA	Serum	31%	
Fecal marker	Fecal calprotectin	Feces	Relatively high sensitivity	This marker reflects the overall level of intestinal inflammation and may aid in the diagnosis of suspected CD (Ribaldone et al., 2020; Hedin et al., 2019).
Salivary marker	Salivary calprotectin	Saliva	Research stage	Although it is a potential non-invasive biomarker, its clinical value remains uncertain, as it may be influenced by other oral inflammatory conditions (Rodrigues et al., 2025).
	Salivary ASCA IgA	Saliva	Research stage	Potential predictive value: In patients with OFG, elevated levels of this antibody may indicate an increased risk of subsequent progression to CD (Savage et al., 2004).

This table provides an overview of the biomarkers used for diagnosing UGI-CD.

The hypothesis that OFG may represent an early manifestation of CD is supported by reports indicating that a substantial proportion of patients with OFG subsequently develop CD. In a study carried out by W. Savage et al., intestinal involvement was predicted by analyzing salivary IgA levels, salivary IgA antibodies against *S. cerevisiae*, and serum IgA antibody levels in patients with OFG. These antibody titers may serve as biomarkers of concurrent intestinal disease and may facilitate the early identification of patients at risk of developing CD. Nevertheless, in the absence of direct intergroup statistical comparisons, it remains uncertain whether the observed differences between the two groups are statistically significant (Savage et al., 2004). Biopsy examination revealed that granuloma is a histological marker of OFG and OCD. In OCD, the incidence of granulomatous inflammation is very high (between 75% and 100%), and characteristic lymphocyte accumulation may help distinguish OFG from OCD (Zbar et al., 2012). In CD, Th1-predominant CD4^+^ lymphocyte responses predominate, whereas Th2-predominant CD4^+^ lymphocyte responses have been detected in biopsy specimens from patients with OFG (Zbar et al., 2012). This is a distinguishing factor that further differentiates it from OFG.

Additionally, isolated oral lesions without intestinal manifestations of CD may be difficult to attribute to CD at diagnosis, and some experts recommend noninvasive examinations. Measurement of intestinal inflammatory markers can improve the diagnostic accuracy of serologic assays. Compared with healthy individuals, patients with CD have significantly higher fecal calprotectin levels, which may aid in the evaluation of suspected CD (Ribaldone et al., 2020; Hedin et al., 2019). Furthermore, a study on salivary calprotectin by Cláudio Rodrigues et al. found that despite its potential as a non-invasive biomarker, its value in the clinical management of CD remains unclear due to its susceptibility to other inflammatory factors (Rodrigues et al., 2025). In combination with endoscopic examination, this may help distinguish OCD from oral granulomatosis. Infectious etiologies must also be excluded, particularly tuberculosis, atypical *mycobacterium* infection, stage III syphilis, and rare fungal infections (cryptococcus and histoplasmosis) (Laube et al., 2018). Because there is no clear or anticipated pattern of oral presentation for intestinal IBD, the diagnostic process should be individualized.

### 2.3 Treatment

The therapeutic goals for patients with IBD and oral lesions are to relieve pain, promote lesion healing, and prevent secondary infections. Medication therapy includes local or systemic corticosteroids, immunosuppressive agents, and biological agents (especially anti-TNF treatment) (Bokemeyer et al., 2018). Among these therapies, systemic corticosteroids and maintenance therapy with thiopurine are considered first-line therapies (Agi et al., 2022). Treatment schedule should be based on the severity of illness, site and number of lesions (Muhvić-Urek et al., 2016).

In a case study by Kim et al., intralesional administration of triamcinolone for oral lesions improved the quality of life by improving symptoms associated with the disease. Owing to the small sample size and the absence of a control group, the results are not generalizable to the wider population of patients presenting with oral lesions (Kim and Lee, 2010). Nonpharmacologic therapy, including ozone therapy and low-intensity laser therapy, may be used to relieve pain and speed up the healing of lesions (Dharmavaram et al., 2015; Albrektson et al., 2014). If there is no improvement in the patient’s symptoms, systemic administration of corticosteroids, immunosuppressants, and thalidomide may be necessary (Katsanos et al., 2015). Biologic medications, especially infliximab, have been demonstrated in multiple studies to be effective in patients who are resistant to or dependent on standard treatments (particularly corticosteroids) (Agi et al., 2022). However, if erythema multiforme or Stevens-Johnson syndrome develops, biologic therapy should be discontinued promptly (Salama and Lawrance, 2009; Edwards et al., 2013).

The use of immunosuppressants can easily lead to secondary infections, with candidiasis being the most common. For oral candidiasis, topical treatment options include nystatin, amphotericin B, miconazole, fluconazole, ketoconazole, and clotrimazole. In some cases, systemic fluconazole, itraconazole, or ketoconazole may be required (Garcia-Cuesta et al., 2014).

Patients with CD should be promptly referred to the Department of Stomatology for appropriate treatment. Implementing a clinical care pathway that reduces the systemic inflammatory response by stabilizing oral lesions is essential. Additionally, patients with gingival involvement should undergo regular dental examinations to maintain good oral hygiene. Additionally, for patients with gingival involvement, regular dental checkups to maintain good oral hygiene are important. If clinically indicated, tooth extraction or gingivectomy can be performed to improve patient comfort, provided that there are no contraindications (Laube et al., 2018; Živić et al., 2023).

### 2.4 Underlying mechanism

While sharing the canonical immunogenetic background, the pathogenesis of OCD is distinguished by the unique ecology of the oral microenvironment. Accumulating evidence highlights the pivotal role of salivary microbiota dysbiosis. A relative enrichment of the genera, including *Streptococcus*, Prevotella, *Haemophilus*, and Veillonella, has been observed in patients with OCD, which may serve as the local trigger for the mucosal immune response. This site-specific microbial alteration, rather than a simple recapitulation of gut dysbiosis, may explain why the oral cavity becomes a primary site of manifestation in a subset of patients (Said et al., 2014).

## 3 Crohn’s disease of the esophagus

Esophageal involvement in CD was first described by Franklin and Taylor in 1950 (Franklin and Taylor, 1950). Esophageal involvement is the least common type of UGI tract involvement, and the reported incidence of esophageal CD in adults ranges from 0.3% to 10% (Feagans et al., 2008). Studies in children with CD have reported that the frequency of esophageal involvement ranges from 5% to 17% (Kővári and Pai, 2022). This difference likely stems from the fact that most pediatric gastroenterology guidelines recommend routine upper gastrointestinal endoscopy at the time of diagnosis. In a series of studies conducted at the Mayo Clinic, more than 12,367 patients with CD were evaluated, and only 24 (0.12%) had esophageal involvement; none had disease confined to the esophagus (De Felice et al., 2015).

### 3.1 Manifestation

Patients with esophageal CD may present with signs and symptoms of esophageal involvement. Most patients are clinically asymptomatic or have mild symptoms, with non-specific UGI symptoms similar to those of gastroesophageal reflux disease (Vale Rodrigues et al., 2020). The most common symptom is dysphagia, followed by odynophagia, heartburn, and retrosternal pain (Monteiro et al., 2017). In a 2012 study, Wang proposed that the progression of esophageal CD could be divided into three stages. The first phase consists of inflammation, edema, erosion, and linear ulceration, with no apparent clinical signs. The second phase develops into a restrictive disease with a mucous bridge clinical. In the last phase, the patient develops progressive dysphagia, odynophagia, vomiting, weight loss, and severe complications resulting from fibrosis and fistulae (Wang et al., 2012).

### 3.2 Diagnosis

The distal (29%) and middle (29%) esophagus are the most frequently involved regions in esophageal CD (De Felice et al., 2015). The diagnosis of esophageal CD includes esophageal-specific symptoms, a history of extraesophageal CD, endoscopic findings, and histopathologic characteristics (Vale Rodrigues et al., 2020).

**FIGURE 1:**
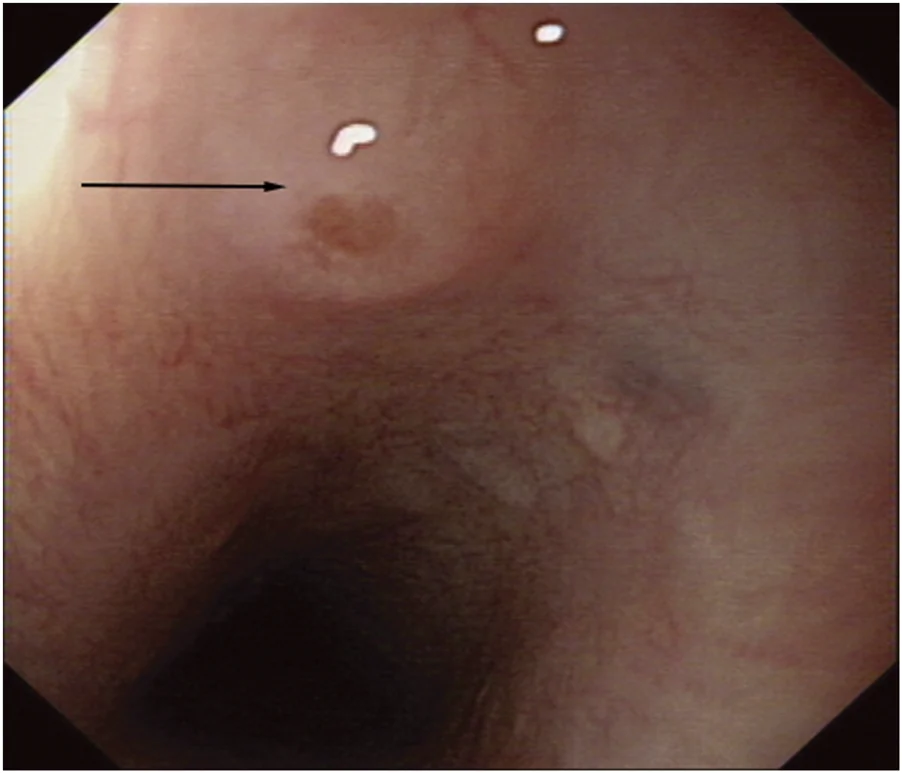
There is a focal, mildly elevated lesion in the esophageal mucosa characterized by surface roughness, congestion, and erosion, highly suggestive of active inflammation (arrow).

The endoscopic appearance is nonspecific. In mild cases, endoscopy typically reveals scattered esophageal erosions (Figure 1). In moderate cases, aphthoid lesions and ulcers are intermingled, and the lesions frequently show a longitudinal distribution (Nomura et al., 2017). In the later stages, a cobblestone appearance, esophageal wall rigidity, strictures, and fistula formation may be observed (Schwartzberg et al., 2019).

Histological examinations are usually nonspecific; biopsy specimens demonstrate features of chronic inflammatory disease but do not identify its underlying cause. The most consistent finding is a lymphocytic infiltrate in the laminapropria, with or without granulomas (Decker et al., 2001). The characteristic histopathologic feature of esophageal CD is the presence of noncaseating granulomas, which are more likely to be located in the submucosal and muscular layers. Because biopsy specimens are typically superficial, obtaining representative tissue samples may be challenging. Therefore, the presence of granulomas is not absolutely necessary for diagnosis (Wang et al., 2012).

Additionally, lymphocytic esophagitis is primarily seen in CD, and its histological feature is an increase in intraepithelial lymphocytes (>20 in one high-power field), with mucosal damage and no granulocytes. Eosinophilic esophagitis (EoE) is characterized by an increased eosinophil count in the esophageal epithelium (≥15 eosinophils/high-power field). Several studies have indicated a fivefold increase in the rate of EoE in IBD (Uchida et al., 2024; Kővári and Pai, 2022; Moore et al., 2021; Limketkai et al., 2019). Although the association between lymphocytic esophagitis and CD has been reported more frequently in pediatric patients, its predictive value appears to be lower in adults. However, the relationship between IBD and lymphocytic/eosinophilic esophagitis requires further investigation to improve understanding of the underlying association (Kővári and Pai, 2022).

Due to the lack of specificity in the diagnosis of esophageal CD, it is necessary to differentiate it from other diseases that may involve the esophagus when diagnosing esophageal CD (Table 3) (Feagans et al., 2008; Debi et al., 2014).

**TABLE 3:** Differential diagnoses of esophageal CD.

Gastroesophageal reflux disease	Esophageal cancer	Viral infections (HSV or CMV)	Drug-induced esophagitis
Sarcoidosis	Tuberculosis	Disseminated fungal diseases	Behçet’s syndrome
Chronic granulomatous disease	Corrosive esophagitis	Esophagitis caused by radiotherapy and chemotherapy	Eosinophilic esophagitis
Epidermolysis bullosa	Metastasis of lung cancer	Lymphoma	Syphilis

This table shows the differential diagnoses of esophageal CD, all listed diseases are independent differential diagnoses.

### 3.3 Treatment

Since esophageal CD is relatively rare, limited data are available regarding appropriate treatment strategies; therefore, no standardized treatment protocol has been established.

According to the European Crohn’s and Colitis Organisation (ECCO) consensus recommendations for the diagnosis and treatment of CD, mild esophageal CD requires treatment with proton pump inhibitors (PPIs), whereas severe or intractable conditions requires additional systemic corticosteroids or anti-TNF therapy. The guidelines indicate that, due to the lack of controlled trials for individualized therapy, most physicians incorporate PPIs to routine treatment. Furthermore, because of the poorer prognosis associated with esophageal CD, the threshold for initiating anti-TNF therapy is lower than that for CD at other anatomical sites (Gomollón et al., 2017).

A retrospective study by Jacob Lui et al. demonstrated that first-line anti-TNFα therapy, was associated with better clinical and endoscopic outcomes than second-line or third-line (e.g., anti-IL-12/IL-23 drugs) biological therapies (Table 4). Given the limited sample size and the absence of randomized controlled trials and prospective studies, large-scale prospective research is required to comprehensively compare the efficacy of different biologic classes for the management of esophageal CD (Lui et al., 2021). Immunomodulating agents, including 6-mercaptopurine, azathioprine, cyclosporine, and thalidomide, are administered to refractory or steroid-dependent patients (Laube et al., 2018; De Greef et al., 2013).

**TABLE 4:** Comparison of the therapeutic effects of biological agents for esophageal CD.

Category of biological agents	Anti-TNFα	Anti-integrin	Anti-IL-12/IL-23
Representative drug	Infliximab Certolizumab Adalimumab	Vedolizumab Natalizumab	Ustekinumab Brazikumab
Clinical response	96.8%	No significant difference	70.0%
Endoscopic response	94.7%	57.1%	57.1%
Patients	18	13 (8 (62%) were previously on anti-TNFα agents)	10 (5 (50%) of these patients were previously treated with anti-TNFα agents and 3 (30%) with anti-integrin agents)
Remarks	It works best as a first-line treatment	The endoscopic response is poor, and it is often used as second-line or third-line treatment	Both the clinical and endoscopic responses are poor, and it is mostly used for refractory cases

This table provides a comparison of the therapeutic effects of biological agents for esophageal CD. The source of the statistical data can be found in the preceding text (Lui et al., 2021).

Endoscopic dilation or surgery may be required for patients with severe disease or disease-related complications (Pimentel et al., 2019; Feagans et al., 2008). In a study by Yan Jia et al., endoscopic radial incision combined with local dexamethasone injection was used to treat CD-induced esophageal stenosis, representing a potential surgical alternative for this condition (Jia et al., 2019). Esophageal resection may be necessary for patients with fistulas or extensive strictures that are refractory to medical therapy and/or endoscopic dilation (Schwartzberg et al., 2019; Reynolds and Stellato, 2001).

### 3.4 Underlying mechanism

The striking epidemiological and immunological overlap with EoE provides a unique perspective on esophageal CD, an association that has not been observed at other UGI sites. The increased risk of CD among patients with EoE suggests the presence of shared or convergent pathogenic pathways, potentially involving type 2 immune responses (Reddy et al., 2024). The hygiene hypothesis, gut microbiome dysbiosis, changes in environmental exposures, and the declining prevalence of *Helicobacter pylori* infection have been proposed to explain the increasing incidence of EoE. In addition, genome-wide association study (GWAS) has identified susceptibility loci specific to EoE, including TSLP, CAPN14, c11orf30/EMSY, STAT6, and ANKRD27. These mechanisms may predispose a subset of patients to the development of manifestations of esophageal CD (Uchida et al., 2024; Spechler, 2018; Sleiman et al., 2015).

Whether the pathogenic mechanisms associated with EoE are involved in the development of esophageal CD remains unclear and warrants further investigation. A deeper understanding of whether esophageal CD represents a distinct immunophenotype or a severe form of luminal CD with secondary eosinophilic infiltration could have profound implications for treatment.

## 4 Gastroduodenal Crohn’s disease

Clinically significant gastroduodenal involvement occurs in 0.5%–4% of patients with CD. Most patients have involvement of the distal small intestine, the large intestine, or both. Approximately one-third of patients with gastroduodenal CD have no small or large intestinal involvement at diagnosis but subsequently develop distal intestinal disease over time (Reynolds and Stellato, 2001; Kefalas, 2003). The most frequent pattern of involvement affects the stomach and duodenum contiguously, with approximately 60 percent of cases presenting with lesions in the stomach, pylorus, and proximal duodenum (Kefalas, 2003; Nugent and Roy, 1989). A slight male predominance has been reported, with a male-to-female ratio of 1.2:1. The condition occurs in both adults and children, with a mean age at diagnosis of 30–40 years (Reynolds and Stellato, 2001; Wagtmans et al., 1997).

### 4.1 Manifestation

The vast majority of patients with CD show no signs of gastroduodenal involvement, and the prevalence of symptomatic disease is less than 4%. The most common symptoms are nonspecific symptoms, including nausea, dyspepsia, anorexia, and upper abdominal pain. The pain is typically postprandial, does not radiate, and is relieved by food or antacids (Schwartzberg et al., 2019). Gastrointestinal strictures may result in premature satiety, post-meal vomiting, weight loss, and rarely hematemesis (van Hogezand et al., 2001). In patients with gastroduodenal CD, gastrointestinal hemorrhage often manifests as chronic anemia, while acute bleeding is very rare (Ingle et al., 2015; Kim et al., 2014). Biliary colic may occur when the ampulla of Vater is involved or when duodenal involvement leads to pancreatitis, although this complication is uncommon (Rao et al., 2008). When the disease is confined to the jejunum or proximal ileum, the primary symptom is abdominal colic (Kim and Kim, 2022).

### 4.2 Diagnosis

The Nugent and Roy criteria constitute the most widely accepted diagnostic standard for gastroduodenal CD. These criteria include: (1) histologic evidence of noncaseating granulomas in the stomach or duodenum, with or without evidence of CD elsewhere; or (2) diagnosis of CD by radiographic or endoscopic examination demonstrating diffuse inflammatory lesions in the stomach or duodenum consistent with CD (Nugent and Roy, 1989).

The diagnosis of gastroduodenal CD requires a high index of clinical suspicion in patients with lower gastrointestinal CD and is confirmed through imaging or endoscopic examination (Kefalas, 2003). Aphthous ulcers may represent early radiographic findings (Levine, 1987), followed by fold thickening, cobblestone formation, pseudodiverticula, and luminal narrowing, which may present as tubularization of the gastric antrum, pylorus, and duodenum (Schwartzberg et al., 2019). An appearance, resembling the postsurgical anatomy after a gastroduodenostomy for peptic ulcer disease, is referred to as the “pseudo-Billroth I appearance” (Pimentel et al., 2019; Schwartzberg et al., 2019; Kefalas, 2003). One distinctive radiologic finding is a funnel-shaped deformity of the diseased gastric antrum and duodenum, known as the “ram’s horn” sign. Dual-contrast radiography may be used to detect strictures, particularly in the later stages of disease (Reynolds and Stellato, 2001). A barium enema is indicated for patients suspected of having a gastrocolic fistula because it is more sensitive for detecting this condition than a UGI series (Kefalas, 2003).

**FIGURE 2:**
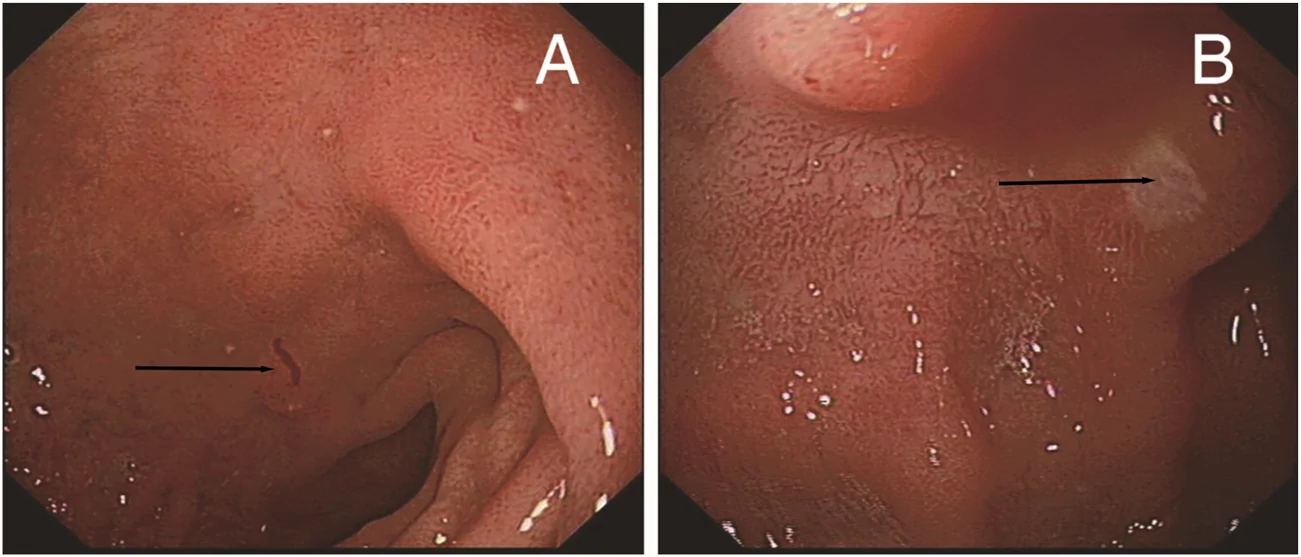
A round ulcer was found near the descending part of the duodenal bulb. The surface was covered with white moss. The mucous membrane was congested and scattered in the hemorrhagic spots (arrow). **(A)** Congestion, erosion and small ulcers are scattered in the duodenal bulb and the descending part (arrow) **(B)**.

Endoscopic examination and biopsy remain the gold standards for diagnosing gastroduodenal CD. These modalities are the most effective methods for detecting early gastric and/or duodenal involvement. Endoscopic features include aphthous ulcers, erosions, patches of erythematous mucosa (Figure 2), thickened folds, fistulas, and a bamboo joint-like appearance (Laube et al., 2018; Kővári and Pai, 2022; Sakuraba et al., 2014; Graca-Pakulska et al., 2023). Notably, the bamboo joint-like appearance of the stomach is a characteristic finding on upper endoscopy in patients with CD. These lesions are characterized by a bulging longitudinal fold intersected by a deep fissure or linear groove and are present in approximately 54% of patients with CD. Histologically, they are characterized by acute, deep, fissuring erosions accompanied by lymphoid aggregates, eosinophilic infiltration, and edema of the peripheral lamina propria. These findings may help identify patients at high risk for CD during gastroduodenoscopy, even before small intestinal involvement becomes apparent (Fujiya et al., 2015). In the duodenum, a cobblestone appearance, polypoid lesions, and notching of the Kerckring folds may also be observed, which are considered characteristic findings (Laube et al., 2018; Wagtmans et al., 1997; van Hogezand et al., 2001).

The histologic features of gastroduodenal CD are usually nonspecific, and multiple biopsies from the stomach and duodenum should be obtained to exclude alternative diagnoses. The prevalence of noncaseating granulomas is between 5% and 83% of all biopsy specimens, with the detection rate of granulomas depending on the location within the gastrointestinal tract and the quality, number, and depth of biopsies obtained (Kefalas, 2003). Granulomas were mostly located in the antrum of the stomach (25%), and then the duodenal bulb (11%) and middle or proximal stomach (6%) (Laube et al., 2018). Granulomas may also be present in several other conditions, including *H. pylori* infection, gastric cancer, peptic ulcer disease, syphilis, gastric lymphoma, sarcoidosis, tuberculosis, eosinophilic gastritis, granulomatosis with polyangiitis, Zollinger-Ellison syndrome, food and suture granulomas, as well as idiopathic isolated granulomatous gastritis. Therefore, the presence of granulomas alone is not a definitive diagnostic criterion for gastroduodenal CD (Pimentel et al., 2019; van Hogezand et al., 2001).

**FIGURE 3:**
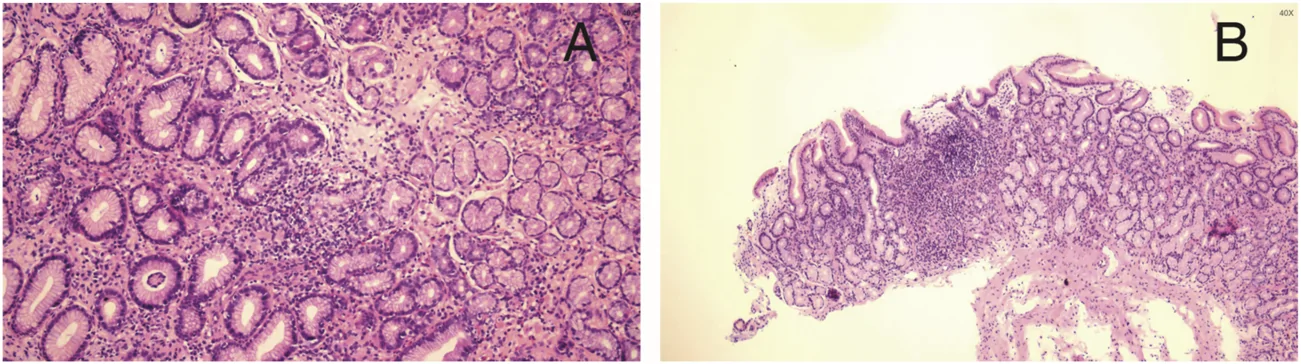
Focally enhanced gastritis (FEG) presents as small clumps of lymphocytes and histiocytes clustered around a small cluster of pits or gastric glands. (arrow) There was mild inflammation around FEG. **(A)** Normal gastric glands were present around FEG. **(B)** (H&E staining).

The most common pathological manifestation of gastroduodenal CD is *Helicobacter* pylori-negative focal patchy gastritis or focally enhanced gastritis (FEG) (Figure 3) with or without granulomas, occurring at a prevalence rate of 76% (Oberhuber et al., 1997). According to the existing literature, *Helicobacter* pylori-negative gastritis is a rare phenomenon. However, a study by Graca-Pakulska et al. found that almost all patients with CD without *H. pylori* infection exhibited pathological features of chronic gastritis. Despite the potential influence of recruitment bias, their findings are in agreement with those previously reported by other investigators. Nevertheless, additional studies are warranted to elucidate the mechanisms underlying these observations (Graca-Pakulska et al., 2023).

FEG, a term coined by Oberhuber et al., is characterized by focal infiltration of CD3^+^ lymphocytes and CD68R+ histiocytes (Oberhuber et al., 1997). According to a retrospective study by Kleoniki Roka et al., 3.4% of patients without IBD had FEG, whereas the prevalence increased to 35.7% among infants with IBD. A significant finding was the presence of sex- and age-related differences, with FEG occurring more frequently in younger patients with IBD and in females, particularly girls younger than 2 years of age. Therefore, in patients with a high clinical suspicion of CD, the presence of FEG may increase the likelihood of diagnosis. However, selection bias in the control group and the lack of blinded histopathologic assessment may have affected the objectivity and validity of the findings, warranting further investigation (Roka et al., 2013). In published data on adults, FEG was common in adult CD (43%) but less common in ulcerative colitis (29%) (Parente et al., 2000; Lin et al., 2010).

Other histological features include mucosal edema, crypt abscesses, lymphoid aggregates, erosions, ulceration, abnormal villous architecture, and fibrosis extending into the muscle (Kefalas, 2003).

### 4.3 Treatment

As gastroduodenal CD is frequently associated with distal disease, treatment typically also targets concomitant small intestinal and colonic involvement. Therefore, gastroduodenal CD is typically treated with the same medications used for CD affecting other regions of the gastrointestinal tract.

PPIs are commonly used for UGI-CD and are effective for symptom relief for nonobstructive disease, as well as in limiting further acid-related mucosal damage in patients with established or suspected gastroduodenal CD (Mottet et al., 2005; Tremaine, 2003). If peptic ulcer disease and *H. pylori* are present, treatment must be administered (Schwartzberg et al., 2019). Although data are scarce, most patients receive standard CD therapies, including 5-aminosalicylic acid, azathioprine, 6-mercaptopurine, methotrexate, and corticosteroids (Reynolds and Stellato, 2001). The prodrug sulfasalazine is activated only after reaching the terminal ileum or colon. Consequently, it is generally ineffective for UGI-CD and may even exacerbate symptoms, thereby limiting its utility in this setting (Schwartzberg et al., 2019; Valori and Cockel, 1990).

For patients with persistent symptoms despite corticosteroid therapy or those requiring maintenance therapy, an immunomodulatory agent, such as azathioprine or 6-mercaptopurine, may be added to the treatment regimen; however, evidence supporting this approach remains limited (Mottet et al., 2005; Goyal et al., 2014). According to a retrospective study by Miehsler and colleagues, 7 out of 12 patients with duodenal CD treated with prednisone and azathioprine were able to discontinue prednisone after 4 to 6 months. Given the small sample size and the lack of validation in large-scale cohorts, larger prospective studies are required to better evaluate its efficacy (Miehsler et al., 2001).

Although the use of infliximab in distal CD has been well established, its role in gastroduodenal CD remains uncertain and has only been evaluated in a few small studies. In a case report by You Lim Kim et al., a man with Crohn’s ileocolitis underwent endoscopic examinations that revealed deep ulcers with deformity in the duodenal bulb, which did not heal after 1 year of treatment with azathioprine, mesalamine, and a proton pump inhibitor. Subsequent treatment with infliximab resulted in complete resolution of the refractory duodenal ulcers, as confirmed by follow-up endoscopy, without complications, suggesting that infliximab may represent a safe and effective treatment option in a similar clinical setting. Because only a limited number of small studies have evaluated its efficacy, large-scale studies are needed to establish the role of infliximab in the treatment of gastroduodenal CD and to determine whether it reduces severe complications and the need for surgery (Kim et al., 2014).

**FIGURE 4:**
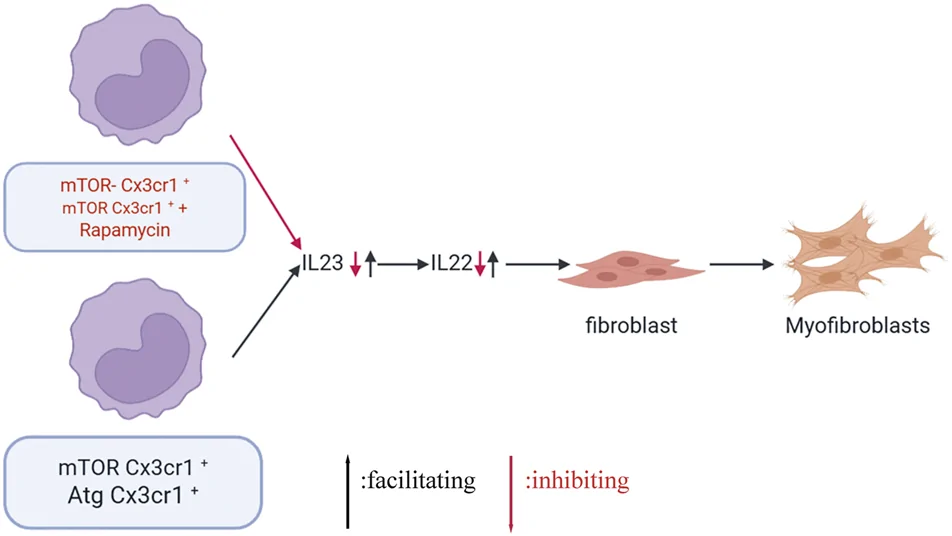
Intestinal damage enhances the IL-23/IL-22 axis by mTOR/autophagy, thereby facilitating the development of fibrosis. Pharmacological inhibition of mTOR by rapamycin or gene deletion of mTOR in Cx3cr1 + mononuclear phagocytes attenuates the induction of IL-23/IL-22 and the resulting fibrosis.

In a study by Mathur et al., rapamycin suppressed the CX3CR1 + mTOR/autophagy pathway and enhanced the IL 23/IL 22 axes in mononuclear phagocytic cells, thereby reducing intestinal fibrosis (Figure 4) (Mathur et al., 2019). In a prospective pilot study by Min Zhong et al. on rapamycin for UGI stenosis, the intention-to-treat population initially included 15 patients; however, one patient with upper gastrointestinal tract involvement and two with lower gastrointestinal tract involvement was subsequently excluded because of adverse events. All patients with UGI strictures (5/5 with gastric and duodenal lesions) achieved clinical remission within 6 months of rapamycin therapy, with an average time of 4.4 months, whereas all lower gastrointestinal stricture patients did not achieve clinical remission. At a median follow-up time of 49 months, three patients maintained long-term treatment success with no relapse for over 1 year. One patient experienced clinical relief of UGI obstructive symptoms after taking rapamycin for 6 months but developed a rectovaginal fistula 16 months after discontinuing treatment. These findings suggest that rapamycin may have a therapeutic role in patients with UGI-CD-related fibrotic stenosis; however, additional controlled studies are needed to confirm these findings (Zhong et al., 2020).

The prevalence of CD-related UGI strictures is less than 4%, and these strictures may complicate the clinical course of the disease (Nugent and Roy, 1989). Endoscopic interventions may be used to manage fibrostenotic strictures of the stomach and duodenum before surgical intervention. Several studies have demonstrated that endoscopic balloon dilation (EBD) can effectively serve as an alternative to surgery for the treatment of fibrotic ileocecal strictures (Rieder et al., 2016; Bettenworth et al., 2017). However, evidence regarding the efficacy of EBD for UGI-CD-associated strictures remains limited (Singh et al., 2017).

In a study by Bettenworth et al., involving 94 patients treated with EBD, the technical success rate was 100%, with a short-term clinical success rate of 87%. The complication rate was 2.9%, and no patient required surgical intervention. Over a median follow-up period of 23.1 months, 70.5% of patients experienced recurrent symptoms, 59.6% required repeat dilation, and 30.8% required surgery. Asian ethnicity and disease location in the jejunum or proximal ileum were identified as factors associated with an increased need for re-dilation. The presence of prestenotic dilation was identified as a risk factor for earlier surgical intervention. As the present study is a meta-analysis of previously reported data, heterogeneity in study designs and clinical management practices may have contributed to bias and influenced the overall findings. Therefore, future research with a prospective design, standardized treatment protocols, and uniform nursing models is required to confirm these observations (Bettenworth et al., 2019).

Surgery remains the only treatment option for those with persistent symptoms, especially obstruction, fistulae, or pain (Schwartzberg et al., 2019). Gastric outlet obstruction (83%), refractory pain (11%), and hemorrhage (5%) are the most common indications for surgery in the management of CD (Desmedt et al., 2024). Other indications include fistula formation and abscess formation. To determine the most appropriate surgical procedure, patients should undergo upper gastrointestinal endoscopy, small-bowel radiography, and colonoscopy to assess the extent of CD (Reynolds and Stellato, 2001; Kefalas, 2003).

The most common surgical treatments for gastroduodenal CD include strictureplasty and bypass procedures, such as gastrojejunostomy, duodenojejunostomy, and gastroduodenostomy. Gastrojejunostomy combined with highly selective vagotomy preserves the autonomic nerve supply to the small intestine and is an effective strategy for reducing complications. While vagotomy reduces the risk of anastomotic ulceration, it is associated with an increased incidence of diarrhea (Pimentel et al., 2019; Kefalas, 2003). Resection has higher morbidity and mortality rates than bypass surgery; therefore, resection is no longer the preferred surgical approach (Schwartzberg et al., 2019).

Various surgical methods have different effects and side effects. Strictureplasty remains an effective option for patients with obstructive duodenal CD, with a lower risk of Dumping Syndrome, Peptic Ulcer, and Biliary Reflux Gastritis than with Bypass Surgery. Nevertheless, this approach has been associated with higher rates of postoperative complications, including intraoperative injury, intra-abdominal infection, restenosis, and reoperation (Moon et al., 2020; Laube et al., 2018; Schwartzberg et al., 2019; Orrell et al., 2021). Yamamoto et al. retrospectively reviewed 13 patients who underwent strictureplasty for obstructive duodenal CD and found that 9 of the 13 patients (69.2%) required reoperation. Given the limited and retrospective nature of the available literature, there is currently no clear consensus regarding which surgical approach is superior (Yamamoto et al., 1999).

A meta-analysis by Campbell et al. compared traditional strictureplasty techniques (Heineke-Mikulicz and Finney) with several alternative approaches and found that the latter were associated with reductions in both acute and long-term complications. Nonetheless, one potential limitation of this analysis is the inherent risk of selective inclusion of studies demonstrating favorable outcomes (Campbell et al., 2012). Similarly, a meta-analysis by Yamamoto et al. confirmed the efficacy of strictureplasty for jejunal CD; however, evidence supporting its use for duodenal involvement remains limited (Yamamoto et al., 2007).

Due to the limited data available in the literature, there is no clear consensus regarding the optimal surgical approach (Yamamoto et al., 2007; Worsey et al., 1999). However, strictureplasty is more appropriate in certain specific situations, such as short-segment strictures with limited edema or in patients with short bowel syndrome (Laube et al., 2018). Compared with open surgery, laparoscopic gastrojejunostomy has the advantages of fewer postoperative complications, faster postoperative recovery, and shorter hospital stay (Salky, 2003).

Several reports have described patients with CD and ampullary involvement associated with pancreatitis and cholangitis, and management may include medical therapy for CD and endoscopic stenting. In selected patients, pancreaticoduodenectomy may be necessary to ensure unobstructed biliary drainage, prevent recurrent episodes of pancreatitis, and treat severe, refractory abdominal pain (Schwartzberg et al., 2019; Tremaine, 2003; Navaneethan et al., 2011).

### 4.4 Underlying mechanism

*Helicobacter pylori* can activate various components of the innate immune system. Studies have indicated that infection with more virulent strains of *H. pylori* (such as CagA-positive strains) may increase the risk of autoimmune diseases, suggesting a potential link between these infections and intestinal disorders like CD. However, the incidence of ulcerative colitis and CD is lower among individuals infected with *H. pylori*. In other words, *H. pylori* infection may serve as a protective factor against the development of IBD (Fujimori, 2021).

Gastroduodenal involvement is a clinical manifestation of CD, and its genetic basis is closely related to that of CD as a whole. Studies have shown that gastroduodenal CD is strongly associated with biallelic variants of the NOD2/CARD15 gene, particularly L1007P homozygosity (Mardini et al., 2005).

## 5 Recapitulation

Reported prevalence rates for UGI-CD involvement at individual anatomical sites vary considerably across studies, largely reflecting differences in diagnostic criteria, biopsy strategies, and patient populations. To facilitate a structured comparison, the prevalence estimates are summarized by anatomical site and stratified according to population type and diagnostic approach (Table 5).

**TABLE 5:** Summary of prevalence rates of UGI-CD by anatomical site.

Anatomical site	Population	Prevalence	Diagnostic method	Influencing factors
Oral cavity	Adults	5%–50%	Clinical oral examination and tissue biopsy.	Whether the assessment was performed by a dental specialist (Rowland et al., 2010; Lankarani et al., 2013).
	Children	Up to 48%	Multidisciplinary evaluation (pediatric gastroenterology and oral medicine).	Children have a higher rate of routine UGI endoscopy with biopsy and a significantly higher detection rate than adults (Pittock et al., 2001).
Esophagus	Adults	0.3%−10%	UGI endoscopy and tissue biopsy.	Most adult guidelines do not routinely evaluate the esophagus initially, potentially leading to missed Dx in asymptomatic patients (Feagans et al., 2008).
	Children	5%–17%	Routine initial UGI endoscopy with biopsy.	Mandatory esophageal biopsy in pediatric practice yields a significantly higher detection rate than that in adults (Kővári and Pai, 2022).
Gastroduodenal	Adults	0.5%–4%	UGI endoscopy with multi-site biopsy and histopathology.	The vast majority of patients are asymptomatic (Reynolds and Stellato, 2001; Kefalas, 2003).
	Children	The trend is similar to that in adults.	Routine pediatric screening improves the detection rate.	There is a lack of large-scale pediatric-specific data (Kim and Kim, 2022).

This table provides a summary of UGI-CD prevalence rates by anatomical site, stratified by population and diagnostic method, with an analysis of influencing factors.

UGI-CD often presents with insidious and nonspecific clinical manifestations involving the oral cavity, esophagus, and gastroduodenum. These manifestations may include painful oral ulcers, reflux-like esophageal symptoms, and epigastric pain or nausea associated with gastroduodenal involvement (Trost and McDonnell, 2005; Monteiro et al., 2017; Schwartzberg et al., 2019). Such manifestations can easily be mistaken for more common disorders, leading to diagnostic delays. In severe cases, complications such as obstruction, strictures, and fistula formation may occur.

**FIGURE 5:**
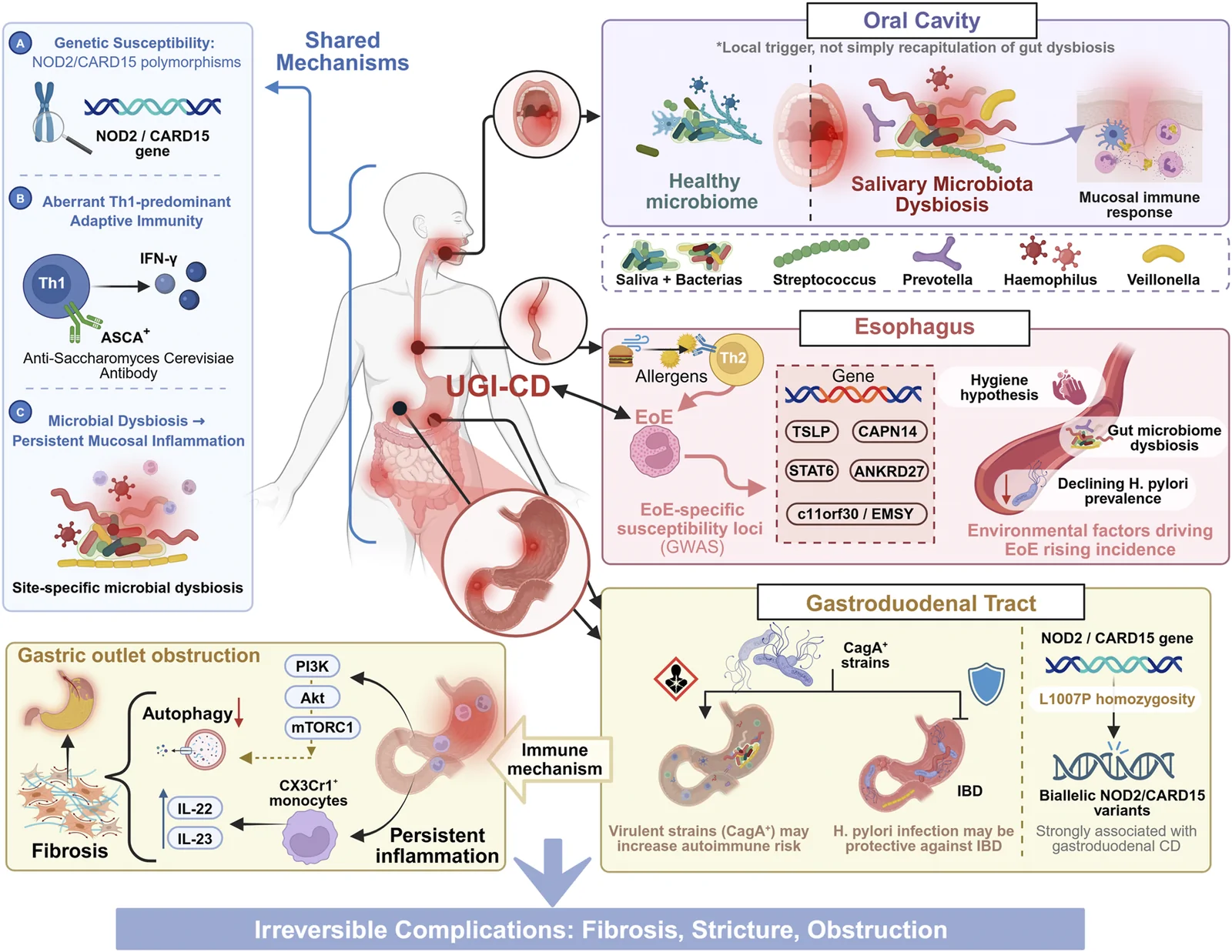
Upper gastrointestinal Crohn’s disease (UGI-CD) shares core mechanisms including NOD2/CARD15 genetic susceptibility, Th1-biased immune abnormality and regional microbial dysbiosis, with site-specific immune and microbial features across the oral cavity, esophagus and gastroduodenum. Chronic inflammation activates IL-23/IL-22 signaling and mTOR/autophagy dysregulation, leading to fibrosis and irreversible obstructive complications.

At the pathogenic level, UGI-CD shares the core pathogenic mechanisms of distal intestinal CD; however, each anatomical site exhibits distinct microenvironmental characteristics. Genetically, mutations in the NOD2/CARD15 gene are associated with CD pathogenesis, with the L1007P homozygous variant being particularly linked to gastroduodenal involvement (Ingle et al., 2015; Zbar et al., 2012; Mardini et al., 2005). Immunologically, aberrant adaptive immune responses dominated by Th1 polarization are central to disease pathogenesis and are reflected by ASCA positivity and T-cell infiltration (Ribaldone et al., 2020). However, the epidemiological overlap observed between esophageal CD and EoE suggests that Th2 pathways may also participate, reflecting the heterogeneity of immune responses across different segments of the upper gastrointestinal tract (Reddy et al., 2024). Microbially, salivary dysbiosis, characterized by enrichment of *Streptococcus* and Prevotella, may serve as a local trigger in the oral cavity (Said et al., 2014), whereas the prevalence of *H. pylori* infection in gastroduodenal CD is paradoxically lower than that in the general population, suggesting a potential protective role (Fujimori, 2021). Ultimately, persistent inflammation may drive fibrosis through dysregulation of the mTOR/autophagy pathway and activation of the IL-23/IL-22 axis, culminating in irreversible complications such as gastric outlet obstruction (Figure 5) (Mathur et al., 2019).

Diagnosis relies on a multimodal integration of high index of clinical suspicion, endoscopic evaluation, and histopathological confirmation. Endoscopic findings may include aphthous ulcers, cobblestoning, strictures, and characteristic features such as the “bamboo-joint-like” appearance of the stomach (Nomura et al., 2017; Schwartzberg et al., 2019; Fujiya et al., 2015). Serological markers (e.g., ASCA) and fecal calprotectin serve as adjunctive diagnostic tools (Ribaldone et al., 2020; Hedin et al., 2019).

The therapeutic strategy aligns with the principles for distal intestinal CD, aiming to control inflammation and promote mucosal healing. PPIs are often used for symptomatic relief, while systemic corticosteroids and immunosuppressants (e.g., azathioprine) constitute the foundation of treatment. For refractory or severe disease, anti-TNF-α biologic agents (e.g., infliximab) have demonstrated significant efficacy (Table 6). In patients with medication-refractory strictures, endoscopic balloon dilation is the first-line intervention, whereas surgical options (e.g., strictureplasty or bypass procedures) are reserved for complications such as obstruction, fistula formation, or failure of endoscopic therapy (Schwartzberg et al., 2019; Rieder et al., 2016; Bettenworth et al., 2017).

**TABLE 6:** Pharmacological treatment strategies and levels of evidence for UGI-CD.

Specific drug	Main application scenarios	Levels of evidence
PPIs	Used for symptom relief in non-obstructive diseases and to protect the mucosa from acid injury.	Expert consensus/Empirical therapy (Gomollón et al., 2017; Mottet et al., 2005; Tremaine, 2003)
Steroids	Used for short-term induction of remission in moderately to severely active disease.	Recommended by the ECCO guidelines (Gomollón et al., 2017)
Immunosuppressants (6-mercaptopurine, azathioprine, methotrexate)	Maintenance therapy in patients with steroid dependence or refractory disease.	Recommended for maintenance of remission (De Greef et al., 2013; Goyal et al., 2014)
Anti-TNF-α biologics (e.g., infliximab)	First-line biologic agent. Used for refractory esophageal, gastroduodenal, and oral lesions unresponsive to conventional therapy.	Strongest evidence: Supported by multiple studies and guidelines for use in refractory cases (Agi et al., 2022; Lui et al., 2021; Kim et al., 2014)
Other biologics (anti-integrin, anti-IL-12/23)	Used as second- or third-line therapy in patients who have failed or are intolerant to anti-TNF-α treatment.	Limited evidence: mostly retrospective studies, with less clearly established efficacy compared to anti-TNF-α therapy (Lui et al., 2021)
mTOR inhibitors (e.g., rapamycin)	Promising therapy. Targeting fibrosis-related strictures associated with UGI-CD.	Preliminary evidence: small prospective studies have shown efficacy for gastric and duodenal strictures, but not for lower gastrointestinal lesions (Mathur et al., 2019; Zhong et al., 2020)

This table highlights current pharmacological treatment strategies and levels of evidence for UGI-CD.

## 6 Conclusion

Currently, the understanding of UGI-CD remains limited. Existing evidence indicates that this subtype is associated with more aggressive disease behavior, poorer nutritional status, and greater heterogeneity in response to conventional therapies. There remains a lack of standardized endoscopic scoring systems for the upper gastrointestinal tract, as well as high-quality prospective studies. Clinicians should maintain a high index of suspicion, recognize the diagnostic value of early upper gastrointestinal endoscopy combined with multisite biopsy for diagnosis, and individualize treatment using nutritional support, immunosuppression, or biologic agents according to the presence of stricturing or penetrating lesions and the burden of systemic inflammation. Future research should focus on unravelling the genetic, cellular and molecular mechanisms underlying upper gastrointestinal Crohn’s disease, as well as the crosstalk between host immunity and the microbiota. High-quality multicenter studies are required to validate the efficacy of targeted therapies, establish reliable imaging and endoscopic stratification criteria, and develop standardized diagnostic and therapeutic frameworks. These efforts will facilitate the development of precision medicine strategies and improve long-term outcomes for affected patients.
